# Ventriculo-Arterial Coupling Is Associated With Oxygen Consumption and Tissue Perfusion in Acute Circulatory Failure

**DOI:** 10.3389/fcvm.2022.842554

**Published:** 2022-02-23

**Authors:** Stefan Andrei, Maxime Nguyen, Dan Longrois, Bogdan A. Popescu, Belaid Bouhemad, Pierre-Grégoire Guinot

**Affiliations:** ^1^Anaesthesiology and Critical Care Department, Dijon Bourgogne University Hospital, Dijon, France; ^2^Department of Anaesthesia and Intensive Care, University of Medicine and Pharmacy “Carol Davila”, Bucharest, Romania; ^3^LNC UMR1231, University of Burgundy Franche Comte, Dijon, France; ^4^Anaesthesiology and Critical Care Department, Bichat Claude Bernard Hospital and INSERM1148, Paris, France; ^5^Department of Cardiology, Emergency Institute for Cardiovascular Diseases “Prof. Dr. C. C. Iliescu”, Bucharest, Romania

**Keywords:** ventricular-arterial coupling, oxygen delivery, oxygen consumption, fluid therapy, norepinephrine, acute circulatory failure

## Abstract

**Introduction:**

The determination of ventriculo-arterial coupling is gaining an increasing role in cardiovascular and sport medicine. However, its relevance in critically ill patients is still under investigation. In this study we measured the association between ventriculo-arterial coupling and oxygen consumption (VO_2_) response after hemodynamic interventions in cardiac surgery patients with acute circulatory instability.

**Material and Methods:**

Sixty-one cardio-thoracic ICU patients (67 ± 12 years, 80% men) who received hemodynamic therapeutic interventions (fluid challenge or norepinephrine infusion) were included. Arterial pressure, cardiac output, heart rate, arterial (E_A_), and ventricular elastances (E_V_), total indexed peripheral resistances were assessed before and after hemodynamic interventions. VO_2_ responsiveness was defined as VO_2_ increase >15% following the hemodynamic intervention. Ventriculo-arterial coupling was assessed measuring the E_A_/E_V_ ratio by echocardiography. The left ventricle stroke work to pressure volume area ratio (SW/PVA) was also calculated.

**Results:**

In the overall cohort, 24 patients (39%) were VO_2_ responders, and 48 patients had high ventriculo-arterial (E_A_/E_V_) coupling ratio with a median value of 1.9 (1.6–2.4). Most of those patients were classified as VO_2_ responders (28 of 31 patients, *p* = 0.031). Changes in VO_2_ were correlated with those of indexed total peripheral resistances, E_A_, E_A_/E_V_ and cardiac output. E_A_/E_V_ ratio predicted VO_2_ increase with an AUC of 0.76 [95% CI: 0.62–0.87]; *p* = 0.001. In principal component analyses, E_A_/E_V_ and SW/PVA ratios were independently associated (*p* < 0.05) with VO_2_ response following interventions.

**Conclusions:**

VO_2_ responders were characterized by baseline high ventriculo-arterial coupling ratio due to high E_A_ and low E_V_. Baseline E_A_/E_V_ and SW/PVA ratios were associated with VO_2_ changes independently of the hemodynamic intervention used. These results underline the pathophysiological significance of measuring ventriculo-arterial coupling in patients with hemodynamic instability, as a potential therapeutic target.

## Introduction

Acute circulatory failure following cardiac surgery is characterized by an imbalance between oxygen delivery (DO_2_) and oxygen consumption (VO_2_) which results in tissue hypoxia and organ dysfunction (1). The challenge in clinical practice is the identification of parameters that are clinically relevant to become endpoints for titration of interventions. Increasing DO_2_ is an accepted goal for optimization following cardiac surgery, especially if it is decreased (2, 3). However, this increase might not be beneficial in all patients. Thus, a concomitant increase in VO_2_ and DO_2_ could identify those patients for whom DO_2_ increase through therapeutic interventions is most beneficial and cost-efficient.

Complementary to the classical heart-centered pressure-flow model, the ventriculo-arterial coupling (V-A coupling) concept describes the interactions between the ventricles and the large arteries from an integrated pressure-volume relationship (4–6). The left ventricle (LV) and the arterial system are described by their elastances [i.e. ventricular elastance (E_V_), arterial elastance (E_A_)], and V-A coupling is defined by the ratio of E_A_/E_V_ (4). The efficacy and efficiency of the cardiovascular system are the result of regulated interactions between the heart and the vascular system. The optimal hemodynamic intervention in patients with acute circulatory failure would improve efficacy with the lowest energetic cost (high efficiency) for the cardiovascular system (7).

Cardiology studies have demonstrated that V-A coupling may represent a parameter that describes the energetic cost in particular when LV function is altered (8, 9). There is clear evidence that V-A coupling is a hemodynamic parameter associated with patient outcomes (8, 10–13). The relevance of V-A coupling as a parameter of hemodynamic optimization in patients with acute circulatory failure could be related to the fact that this represents a parameter of cardiovascular efficiency whereas the classical hemodynamic parameters are exclusively parameters of cardiovascular efficacy (2, 3). Guidelines regarding the implementation of V-A coupling in clinical use are available for cardiologists, particularly in the context of heart failure (14). However, studies evaluating V-A coupling-based hemodynamic algorithms in the setting of ICU acute circulatory failure are lacking.

The present study was designed to investigate the clinical relevance of V-A coupling in critically ill patients. We sought to analyse the effects of two types of interventions: fluid challenge or norepinephrine infusion on systemic oxygenation parameters (as indicators of cardiovascular efficacy) and on V-A coupling (as an indicator of cardiovascular efficiency).

The main objective of this study was to investigate the relationship between E_A_/E_V_ ratio and changes in VO_2_ upon treatment of hemodynamic instability following cardiac surgery. The second objectives were to compare V-A coupling and oxygenation-derived parameters [central venous saturation (ScVO_2_), gap of CO_2_] as predictor of VO_2_ changes following hemodynamic treatment.

## Methods

### Ethics

The study's objectives and procedures were approved by the local independent Ethics Committee (Comité de Protection des Personnes Nord-Ouest II CHU - Place V. Pauchet, 80054 AMIENS Cedex 1). All patients received written information and gave their verbal consent to participate. The present manuscript was drafted in compliance with the STROBE checklist for cohort studies (15).

### Patients

This observational cohort study was performed in a university-affiliated cardiothoracic ICU between 2015 and 2017. We included patients aged 18 or over, under controlled positive ventilation, with hemodynamic instability, for whom the clinician decided to perform a fluid challenge and/or start a norepinephrine infusion. The indications for fluid challenge were: (1) arterial hypotension, defined as systolic arterial pressure (SAP) below 90 mmHg and/or a mean arterial pressure (MAP) below 65 mmHg, and/or (2) stroke volume (SV) variation of more than 10%, and/or (3) clinical signs of hypoperfusion. In the present study, fluid challenge always consisted of a 10-min infusion of 500 ml of lactated Ringer's solution (16). The indication for norepinephrine was persistent arterial hypotension (SAP <100 mmHg and/or MAP <65 mmHg) despite fluid challenge (11, 16). The exclusion criteria were permanent/persistent chronic atrial fibrillation, heart conduction block (type I, II and III atrioventricular blocks, left and right bundle branch blocks), the presence of an active pacemaker, poor acoustic window, more than mild aortic regurgitation, and right heart failure.

### Measurement and Calculation of Left Ventricular Elastance, Arterial Elastance, and Ventriculo-Arterial Coupling

Stroke volume (SV; mL) and cardiac output (CO; l min^−1^) were measured using transthoracic echocardiography performed with a commercially available machine (CX50 ultrasound system Philips Medical System, Suresnes, France) equipped with a S5-1 Sector Array Transducer. The echocardiographic parameters were averaged from five measurements (regardless of the respiratory cycle). E_V_ was estimated at the bedside using the non-invasive single beat method described by Chen et al. (17). This method is based on the assumption that time-variation of LV elastance is not influenced by loading conditions or heart rate. E_V_ was calculated by the formula: E_V_ = (Pd – (E_Nd(test)_^*^ Pes ^*^ 0.9)) / (SV ^*^ E_Nd(test)_). E_Nd(test)_ was obtained from a group-averaged normalized elastance curve value at this same time td (E_Nd(avg)_), baseline LV ejection fraction (LVEF) and the ratio of diastolic to systolic arterial pressure (17). We calculated the coefficient of variation (CV), precision and least significant change (LSC) for E_V_ in ten patients. CV was 7.7% ± 0.6 and LSC was 10.9% ± 0.8. E_A_ was estimated by using the formula E_A_= end-systolic pressure (ESP = 0.9 ^*^ SAP)/SV (18). SAP was measured by using invasive radial artery catheters. In healthy men and women, the mean E_A_/E_V_, E_A_, and E_V_ values measured invasively at rest are respectively 1.0 ± 0.36, 2.2 ± 0.8 mmHg ml^−1^, and 2.3 ± 1.0 mmHg.ml^−1^ (18, 19). An abnormal E_A_/E_V_ ratio was defined as a value >1.36 (19).

The total energy generated by each cardiac contraction is called the “pressure-volume area” (PVA), which is the sum of the external mechanical work exerted during systole (SW) and the potential energy (PE) stored at the end of systole: PVA = SW + PE (19). The PVA has been demonstrated to be linearly related to myocardial oxygen consumption (7, 19). SW is calculated as ESP x SV. Potential energy is calculated as ESP x ((ESV-V_0_)/2) and assumes that V_0_ is negligible when compared with ESV. We calculated total indexed peripheral resistance (TPRi) as TPRi = MAP-central venous pressure (CVP)/cardiac index (mmHg ml^−1^ m^−2^).

### Oxygenation Parameters

We recorded the ventilator settings (tidal volume, plateau pressure and end-expiratory pressure) at baseline. All parameters were measured on arterial and central venous blood gases (Supplementary File 1).

### Study Procedures

Anesthesia and cardiopulmonary bypass procedures were standardized for all patients. During the study period, the patients were mechanically ventilated in volume-controlled mode, with a tidal volume set to 7–9 ml kg^−1^ ideal body weight, and a positive end-expiratory pressure (PEEP) of 5–8 cm H_2_O, and sedated with Propofol (20). Ventilator settings (oxygen inspired fraction, tidal volume, respiratory rate and end positive pressure) were not modified during the study period.

The following clinical parameters were recorded: age, gender, weight, ventilation parameters, and primary diagnosis. After an equilibration period, HR, SAP, MAP, diastolic arterial pressure (DAP), CVP, SV, CO, and arterial/venous oxygen content were measured at baseline.

### Statistical Analyses

In the absence of preliminary data, we designed an observational study with a convenience sample of 61 consecutive patients. Such size could enable to demonstrate a correlation (0.3–0.5) between E_A_/E_v_ ratio and VO_2_ response with a power of 0.8 and alpha error of 0.05. The variables' distribution was assessed using a D'Agostino-Pearson test. Data are expressed as the number, proportion (in percent), mean ± standard deviation (SD) or the median [interquartile range (IQR)], as appropriate. Patients were classified as VO_2_ responders or non-responders as a function of the effect of hemodynamic interventions (fluid challenge or norepinephrine) on VO_2_. VO_2_ response was defined as an increase of more than 15% in the VO_2_. The non-parametric Wilcoxon rank sum test, Student's paired *t*-test, Student's *t*-test, the Mann-Whitney test, and the Fisher's test were used to assess statistical significance, as appropriate. Correlations were tested using Pearson's or Spearman's rank test, as appropriate.

Because we have analyzed several correlated hemodynamic and perfusion variables, we performed an exploratory principal component analysis to avoid collinearity. The principal component analysis transforms correlated variables into uncorrelated variables that may explain VO_2_ changes. A principal component analysis was carried out by including fourteen baseline variables. The VO_2_ changes following therapeutic interventions were included as a supplementary variable. Considering there were some differences between baseline characteristics between VO_2_ responders vs. non-responders in univariate, we performed a multivariate regression logistical model in order to determinate the independent adjusted associations. The conditions of validity of the multivariate model were checked, in order to have at least 5 responders for each variable included in the model. A receiver-operating characteristic (ROC) curve was established for the ability of ScVO_2_, CO_2_ gap, E_V_/E_A_ ratio to predict an increase of more than 15% in VO_2_.

The threshold for statistical significance was set to *p* < 0.05. R software (version 3.5.0) with FactoMineR package was used for all statistical analyses.

## Results

Of the 65 included patients, four were excluded (Supplementary File 2), and so the final study population consisted of 61 subjects (Table 1). At baseline 48 patients (78%) were categorized as uncoupled, with a median E_A_/E_V_ ratio of 1.9 (1.6–2.4) mainly related to abnormally low E_V_ (1.1 (0.9–1.6)), as compared to preserved E_A_ (2 (1.5–2.7)). In the overall population, 31 patients (48 %) were classified as VO_2_ responders. The percentage of VO_2_ responders did not differ between the two groups [16 (48%) out of 33 vs. 15 (54%) out of 28, *p* = 0.799]. Of the 6 patients with mitral regurgitation surgery, 2 were in the group of VO_2_ responders and 4 in the group of VO_2_ non responders (Fisher's test, *p* = 1).

**Table 1:** Characteristics of the study participants on inclusion.

**Variables**	**Overall population (*n* = 61)**
Age (years), mean±SD	67 ± 12
Gender (female), *n* (%)	12 (20)
BMI (kg m^−2^), mean±SD	28.7 ± 5
Comorbidities, n (%)	
- High blood pressure	44 (72.1)
- Heart failure	21 (34.4)
- Right ventricle dysfunction	9 (14.8)
- Diabetes	16 (26.2)
- Dyslipidaemia	28 (45.9)
- Active smoking	22 (36.1)
- COPD	5 (8.2)
- Chronic kidney disease	5 (8.2)
SAPS 2 score, mean±SD	41 ± 14
ASA score, *n* (%)	
- I	1 (1.6)
- II	2 (3.3)
- III	54 (88.5)
- IV	4 (6.6)
Type of surgery, *n* (%)	
- Valvular only	29 (47.5)
- Mitral regurgitation surgery	6 (9.8)
- CABG only	10 (16.4)
- Combined surgery	12 (19.7)
- Other	10 (16.4)
*Ventilation parameters*	
Tidal volume (ml kg^−1^ of predicted body weight); mean ±SD	7.7 ± 0.6
Total PEEP (cmH_2_O), mean±SD	5 ± 1
Patients treated with norepinephrine, *n* (%)	26 (48)
Median dose (mcg Kg^−1^min^−1^), (IQR)	0.08 [0.06–0.18]
Patients treated with fluid expansion, *n* (%)	35 (52)
In-hospital death, *n* (%)	3 (6)

*SD, standard deviation; BMI, body mass index; COPD, chronic obstructive pulmonary disease; SAPS 2, simplified acute physiology score 2; ASA score, American Society of Anesthesiology score; CABG, coronary artery bypass graft surgery; PEEP, positive end expiratory pressure; IQR,25–75% interquartile range*.

### Combined Analysis of the Effects of the Two Therapeutic Interventions on Systemic Parameters

**Figure 1:**
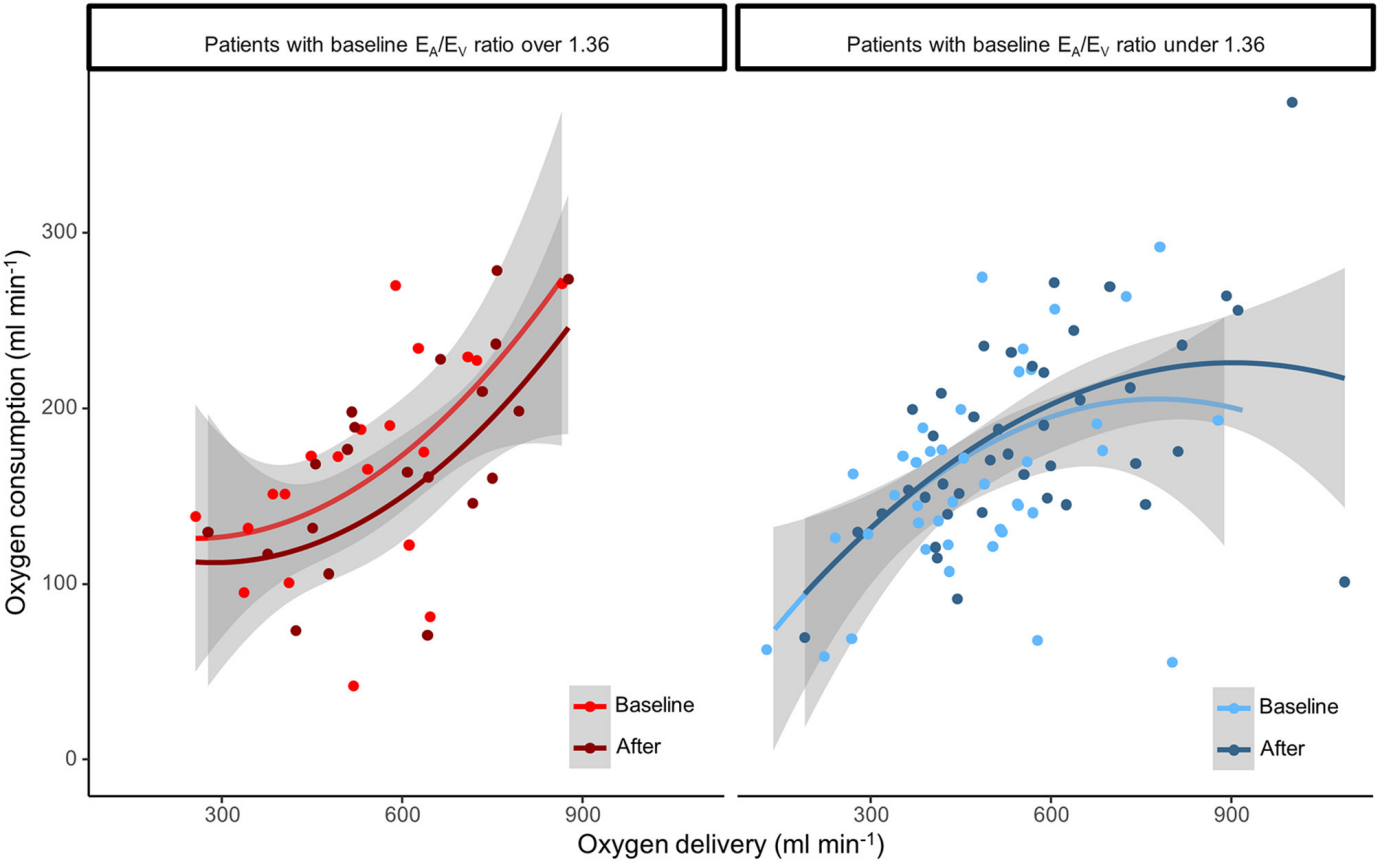
Relationship between oxygen delivery (DO_2_) and oxygen consumption (VO_2_) in uncoupled and coupled patients before and after the hemodynamic intervention.

At baseline, VO_2_ responders had higher E_A_/E_V_ ratio, and lower SW/PVA ratio and VO_2_ than VO_2_ non-responders (Table 2, Figure 1). They also had a lower LVEF. Therapeutic interventions increased SAP, MAP, CO and DO_2_ in the overall population. VO_2_ responders were characterized by an increased SW/PVA ratio, and a decreased HR, and TPRi. VO_2_ non-responders were characterized by an increased E_A_, E_V_, ScVO_2_, TPRi, and a decreased gapCO_2_.

**Table 2:** Comparison of haemodynamic parameters in VO_2_ responders and VO_2_ non-responders. Values are expressed as the mean (SD) or the median (interquartile range).

**Hemodynamic variables**	**VO_2_ responders (***n*** = 31)**	**VO_2_ non-responders** **(***n*** = 30)**	***p value***
HR (bpm)			
Pre	78 (21)	82 (19)	0.410
Post	75 (20)^$^	80 (16)	0.274
SAP (mmHg)			
Pre	99 (19)	91 (14)	0.093
Post	124 (19)^$^	116 (20)^$^	0.333
MAP (mmHg)			
Pre	68 (15)	65 (10)	0.260
Post	84 (14)^$^	80 (12)^$^	0.707
SV (ml)			
Pre	41 (14)	46 (18)	0.413
Post	57 (19)^$^	50 (16)^$^	0.118
CO (L min^−1^)			
Pre	3.2 (1.1)	3.6 (1)	0.159
Post	4.1 (1)^$^	3.8 (0.9)^$^	0.357
TPRi (mmHg ml^−1^ m^−2^)			
Pre	42 (14)	34 (15)	0.056
Post	39 (17)^$^	39 (14)^$^	0.945
E_A_ (mmHg ml^−1^)			
Pre	2.3 (1)	2.1 (1)	0.358
Post	2.2 (0.8)	2.4 (1.1)^$^	0.343
E_V_ (mmHg ml^−1^)			
Pre	1.2 (0.6)	1.3 (0.6)	0.252
Post	1.2 (0.6)	1.6 (0.7)^$^	0.036
E_A_/E_V_			
Pre	2.2 (0.6)	1.6 (0.6)	0.002
Post	2 (0.9)	1.6 (0.5)	0.023
SW/PVA ratio			
Pre	0.55 (0.12)	0.62 (0.11)	0.008
Post	0.62 (0.15)^$^	0.62 (0.12)	0.891
DO_2_ (ml min^−1^)			
Pre	482 (179)	504 (146)	0.603
Post	635 (219)^$^	539 (149)^$^	0.047
VO_2_ (ml min^−1^)			
Pre	132 (54)	180 (53)	0.001
Post	198 (61)^$^	167 (53)^$^	0.041
ScVO_2_ (%)			
Pre	67 (12)	60 (9)	0.01
Post	63 (9)^$^	65 (8)^$^	0.842
GapCO_2_ (mmHg)			
Pre	9 (4)	9 (2)	0.842
Post	9 (4)	7 (5)^$^	0.061
Arterial lactate (mmol l^−1^)			
Pre	1.5 (1.3–2.1)	1.6 (1.3–2.1)	0.882
Post	1.5 (1.2–2.1)	1.7 (1.3–2.1)	0.468
LVEF (%)			
Pre	42 (13)	50 (11)	0.007
Post	46 (12)	49 (9)	0.209

*CO, cardiac output; DO_2_, oxygen delivery; FC, fluid challenge; HR, heart rate; LVEF, lef ventricular ejection fraction; MAP, mean arterial pressure; SAP, systolic arterial pressure; SV, stroke volume; TPRi, total indexed peripheral resistance; VO_2_, oxygen consumption*.

$ *p < 0.05 within groups (pre-/post-FC)*.

### Effects of Fluid Challenge on Systemic Oxygenation Parameters

At baseline, VO_2_ responders had lower VO_2_, gapCO_2_, and higher E_A_/E_V_ ratio, ScVO_2_ than VO_2_ non-responders. Fluid challenge increased SAP, MAP, and SV in VO_2_ responders and non-responders. VO_2_ responders were characterized by an increase in SV and CO decreased TPRi, E_A_, and an increased SW/PVA ratio and gapCO_2_. E_V_ did not change. VO_2_ non-responders were characterized by an increase in SV, ScVO_2_, and a decreased HR.

### Effects of Norepinephrine on Systemic Oxygenation Parameters

At baseline, VO_2_ responders had lower SV, CO, SW/PVA ratio, VO_2_, and higher gapCO_2_, E_A_/E_V_ ratio than VO_2_ non-responders. Norepinephrine infusion increased SAP, MAP, CO and DO_2_ in both groups. VO_2_ responders were characterized by an increased SV and CO, SW/PVA ratio. VO_2_ non-responders were characterized by an increased E_A_, E_V_, ScVO_2_.

### Correlations Between Systemic Oxygenation Parameters (Efficacy) vs. E_A_, E_V_, E_A_/E_V_ Ratio and SW/PVA Ratio (Efficiency) With the Two Therapeutic Interventions

In the overall cohort, changes in VO_2_ were correlated with those in SW/PVA ratio (r = 0.362, *p* = 0.003), E_A_ (r = −0.446, *p* < 0.001), E_A_/E_V_ (r = −0.256, *p* = 0.046), CO (r = 0.495, *p* < 0.001), ScVO_2_ (r = −0.522, *p* < 0.001), TPRi (r = −0.444, *p* < 0.001). The baseline SW/PVA ratio was correlated with DO_2_ (r = 0.339, *p* = 0.004), VO_2_ (r = 0.258, *p* = 0.045), and gapCO_2_ (r = −0.304, *p* = 0.017).

### VO_2_ Responsiveness Prediction

Baseline E_A_/E_V_ was predictive of VO_2_ responsiveness, with an area under the curve (AUC) [95% confidence interval (95%CI)] of 0.76 ([0.62–0.87]; *p* = 0.001). The best cut-off was 2.1 with a gray zone between 1.8 and 2.4. With an AUC [95%CI] of 0.72 [0.59–0.85] (*p* = 0.004), baseline ScVO_2_ was predictive of VO_2_ responsiveness. With an AUC [95%CI] of 0.44 [0.29–0.58] (*p* = 0.05), gapCO_2_ was not predictive of VO_2_ responsiveness. When analyzing patients separately for fluid challenge or norepinephrine infusion, baseline E_A_/E_V_ was predictive of VO_2_ responsiveness in fluid challenge group (AUC: 0.77 [0.59–0.95] (*p* = 0.008) and norepinephrine group (AUC: 0.74 [0.56–0.93] (*p* = 0.045).

These results were confirmed by the multivariate regression model adjusted on differences at baseline (LVEF, ScVO_2_, SW/PVA ratio, E_A_/E_V_ ratio). Baseline E_A_/E_V_ ratio (OR: 3.4 [1.1–10.3], *p* = 0.033) and ScVO_2_ (OR: 1.08 [1.01–1.15], *p* = 0.016) were independently associated with VO_2_ responsiveness, but not the baseline LVEF (OR: 1 [0.93–1.07], *p* = 0.993) and SW/PVA ratio (OR: 0.5 [0–13], *p* = 0.297). When using the principal component analysis, the 3 first principal components explained 61% of the variance (Supplementary Table 1, Supplementary Figures 1, 2). VO_2_ changes were significantly associated with the first (r = 0.31) and the third component (r = 0.52). E_A_, E_V_, E_A_/E_V_, and SW/PVA ratio were variables included in components associated to VO_2_ changes.

## Discussion

The main results of the present study are as follow: (1) most patients for whom fluid challenge or norepinephrine infusion increased VO_2_ had high V-A coupling ratio with lower left ventricle stroke work to pressure volume area ratio (SW/PVA) at baseline; (2) baseline E_A_/E_V_ and SW/PVA ratios were associated with perfusion parameters and VO_2_ changes independently of the therapeutic intervention used.

When analyzing together fluid challenge or norepinephrine infusion, the only common profile is the increase in arterial pressure. VO_2_ responders have an increase in SV, CO and a decrease in TPRi. VO_2_ responder patients were uncoupled before interventions as they adapted to maintain tissue perfusion with a higher energetic cost for the same efficacy (preserving efficacy over efficiency). This was reflected by the lower E_A_/E_V_ ratio in VO_2_-responders. Equally, VO_2_-responder patients had significantly lower SW/PVA values before hemodynamic intervention, which were associated to perfusion parameters. We demonstrated that the E_A_/E_V_ ratio partly explains VO_2_ responsiveness and was independently associated with VO_2_ responsiveness. Both approaches credibly establish at least the statistical relevance of analyzing V-A coupling in patients with hemodynamic instability following cardiac surgery.

### A Pathophysiological Perspective on V-A Coupling

It has been shown that mechanical efficiency is greatest when E_A_ = E_V_ (i.e., E_A_/E_V_ ratio = 1) (4, 6, 21). The patients of the present study were characterized by “normal” E_A_ but much lower than normal E_V_ values, resulting in 78% patients having high V-A coupling ratio. Burkhoff and Sagawa have also shown that the mechanical efficiency of the heart is more sensitive to E_A_, especially when E_V_ is impaired, which is observed at baseline in the patients of the present study (21). “Sacrificing” efficiency to preserve efficacy for a limited period of time is a “physiological choice” observed in athletes (22). Patients with the most severe V-A coupling (i.e., the highest V-A coupling ratio) had the lowest VO_2_ (22). The consequences of long term “sacrificing efficiency,” (i.e., days) for the ICU patients are not known. For instance, the fact that catecholamine use is associated with increased mortality could be an example of deleterious long-term consequences of providing better cardiovascular performance at a high energetic cost (23).

Investigating the effects of two interventions on V-A coupling comes down to answering the question already raised many years ago: how effective is an increase in myocardial performance (i.e., an increase in SV) transmitted to the peripheral circulation (24). This transmission may be mediated by the V-A coupling (24). In this respect, if the increase in cardiac performance is transmitted to the circulation, this should result into opening new vascular beds, and if DO_2_ limits the VO_2_, this should result in an increase in VO_2._ This is what our results demonstrate, linking the increase in cardiac performance with the peripheral circulation through the V-A coupling.

### Clinical Relevance of V-A Coupling in ICU Patients

Cardiologists have already integrated V-A coupling based hemodynamic approach in the treatment of chronic heart failure or arterial hypertension (9, 14, 25). In the ICU, several attempts of hemodynamic optimisation based on V-A coupling perspective were published (25, 26). Few studies have investigated the relationship between V-A coupling on one side and DO_2_ and VO_2_ on the other in ICU patients (10, 27). To the best of our knowledge, this is the first attempt that has specifically focused on VO_2._ V-A coupling has been already demonstrated as a factor limiting patients' adaptability to effort (8). Previous authors have studied the association of V-A coupling improvement and the time course of systemic oxygenation parameters in trauma patients (27, 28). Our results support their findings by demonstrating an association between V-A coupling, SW/PVA ratio, to perfusion parameters and further VO_2_ changes.

Sepsis and septic shock are characterized by different profiles of V-A coupling (i.e., different hemodynamic profiles) for which hemodynamic treatment may differ (29). More than two thirds of sepsis patients are V-A uncoupled (30, 31). The fact that most uncoupled patients showed an increase in VO_2_ after therapeutic interventions could be a further argument for targeting V-A coupling during resuscitation, as marker of tissular perfusion. This therapeutic option might help in better choosing the responsive patients, as a vasopressor-sparing strategy (32). The norepinephrine infusion alters V-A coupling with an increase in Ea >> Ev (30, 33). Targeting primarily the MAP of 65 mmHg might sustain an unnecessary (34) and possibly an unfavorable prolonged energetic state because of increased cardiac afterload (32). Algorithms using the monitoring dynamic arterial elastance were proposed in ICU and they demonstrated that early weaning of norepinephrine may be associated with better tissue perfusion (12, 35). As the dynamic arterial elastance was shown to be correlated with V-A coupling and microcirculation (36, 37), the connection with VO_2_ in our study might contribute with a new puzzle piece. One advantage of V-A coupling is that it can be non-invasively measured at bedside. Contrary to perfusion parameters, it does not require blood sampling. Further randomized study evaluating a hemodynamic strategy based on V-A coupling may confirmed these points.

### Potential Limitations of the Present Study

Firstly, the analysis of two therapeutic interventions can make interpretation of the results difficult. The present objective was not to precisely analyze the individual effect of each therapy. Such demonstrations have been previously published (17). On the contrary, we would like to demonstrate that a hemodynamic approach based on the V-A coupling makes it possible to dispense with the hemodynamic treatment and a detailed analysis of each parameter. The fact that the association between V-A coupling and perfusion parameters was demonstrated in the population as a whole and in each treatment, group reinforces our results. As discussed, we believe that the effects of norepinephrine on VO_2_ may be due to its effects on CO and DO_2_ (38).

Secondly, the VO_2_/DO_2_ relationship is not linear. The VO_2_ responder group has lower values of VO_2_ that are below those in the non-responder group, even after hemodynamic treatment. We believe the lower value of VO_2_ in responder group may not have introduce bias. These observations are in relation with the fact that the hemodynamic response was defined by VO_2_ changes. The methods used to calculate E_V_ and E_A_ can potentially be criticized because we did not use a high-fidelity ventricular pressure catheter (17). We calculated ESP from a radial artery signal, which may differ from the aortic pressure signal. However, radial artery pressure has been reported to provide a good estimate of ESP (39). Although it can be argued that estimation of ESP from the radial artery has not been fully validated, any error in this method would only affect the precision of absolute values of E_A_ and E_V_, but not the E_A_/E_V_ ratio, as the error in end-systolic pressure would be similar. Despite these limitations, non-invasive evaluation of E_V_ and E_A_ was validated against the gold standard method and has been used in cardiac surgery (5–7). In the present study, E_A_ and E_V_ must be considered to be approximations of E_A_ and E_V_. Despite these limitations, non-invasive evaluation was validated against the gold standard method, and have been used in the cardiology and cardiac surgical fields (14).

Thirdly, our findings generalizability is restricted by the low number of patients analyzed in this cohort. Considering the known heterogeneity of intensive care unit (ICU) patients, larger cohorts are required to perform a properly matching on different confounding factors. The clinical relevance of the relationship between V-A coupling and VO_2_ in the context of goal-directed therapy in critically ill patients is still to be validated. The results of well-designed interventional trials, such as the one published by Borlaug et al that used LV afterload reduction, are awaited (40).

## Conclusions

In VO_2_ responders, V-A coupling was characterized by a high E_A_/E_V_ ratio (due to high E_A_ and low E_V_). Baseline E_A_/E_V_ and SW/PVA ratios were associated with VO_2_ changes independently of the hemodynamic intervention used. Measuring V-A coupling may offer a new perspective of hemodynamic optimisation in the ICU by individualizing hemodynamic treatment and by analyzing both the efficacy and efficiency of hemodynamic interventions.

## Data Availability Statement

The raw data supporting the conclusions of this article will be made available by the authors, without undue reservation.

## Ethics Statement

The studies involving human participants were reviewed and approved by (Comité de Protection des Personnes Nord-Ouest II CHU - Place V. Pauchet, 80054 AMIENS Cedex 1). The patients/participants provided their written informed consent to participate in this study.

## Author Contributions

SA, MN, and P-GG conceived the study, analyzed the data and drafted the manuscript. P-GG collected the data. DL, BP, and BB provided a critical review. All authors contributed to the article and approved the submitted version.

## Conflict of Interest

The authors declare that the research was conducted in the absence of any commercial or financial relationships that could be construed as a potential conflict of interest.

## Publisher's Note

All claims expressed in this article are solely those of the authors and do not necessarily represent those of their affiliated organizations, or those of the publisher, the editors and the reviewers. Any product that may be evaluated in this article, or claim that may be made by its manufacturer, is not guaranteed or endorsed by the publisher.
