# Supplementary material for: Ventriculo-Arterial Coupling Is Associated With Oxygen Consumption and Tissue Perfusion in Acute Circulatory Failure
Source: Front Cardiovasc Med. 2022 Feb 23;9:842554. doi: 10.3389/fcvm.2022.842554 (PMC8904883; doi:10.3389/fcvm.2022.842554)

Supplementary Material

***File 1: Oxygenation parameters description***

We recorded the ventilator settings (tidal volume, plateau pressure and end-expiratory pressure) at baseline. All parameters were measured on arterial and central venous blood gases. All pressure monitors were zeroed at the mid-axillary line upon arrival at the ICU, and the position of the tip of the central venous catheter in the upper part of the right atrium was verified by chest radiography.

Arterial and venous blood samples, lactate, haemoglobin concentrations and oxyhaemoglobin saturation were assayed using an automated analyser (ABL800 FLEX®, Radiometer, Bronshoj, Denmark). Arterial (CaO_2_) and venous oxygen contents (CvO_2_) were calculated as follows: CaO_2_ = 1.34 x Hb x SaO_2_ + 0.003 x PaO_2_; CvO_2_ = 1.34 x Hb x ScvO_2_ + 0.003 x PvO_2_, where Hb is the haemoglobin concentration (g dl^-1^), PaO_2_ is the arterial oxygen pressure (mmHg), SaO_2_ is the arterial oxygen saturation (%), PvO_2_ is the venous oxygen pressure (mmHg), and ScvO_2_ is the central venous oxygen saturation (in %). The C(a-v)O2 was calculated as CaO_2_ minus CvO_2_ (ml). PCO_2_ gap was calculated as PcvCO_2_ minus PaCO_2_ (mmHg). C(a-v)O_2_ was calculated as CaO_2_ minus CvO_2_ (ml).

DO_2_ and VO_2_ were calculated as follows: DO_2_ (ml min^-1^) = (CaO_2_ x 10 x CO); VO_2_ (ml min^-1^) = the arteriovenous difference in oxygen content ((*C*(*a* − *v*)*o*_2_) x CO x 10).

**File 2: Study Flow chart diagram.**

**
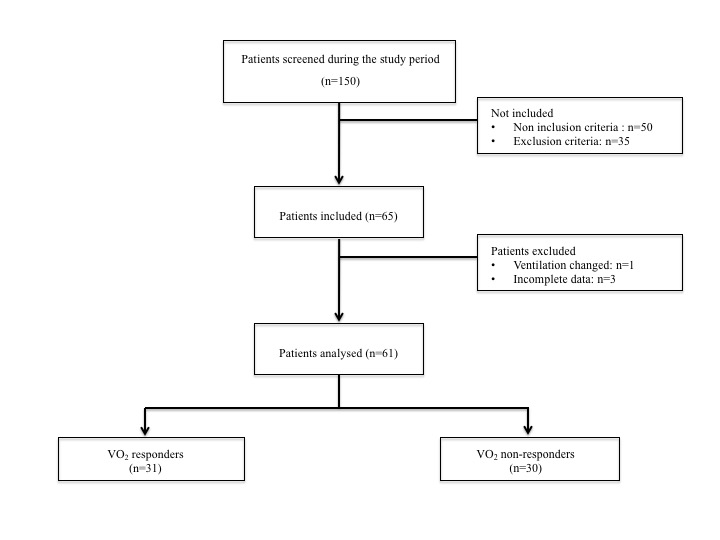
**

**Supplementary Table 1.** Principal components description. **CO**, cardiac output; ***DO_2_***, oxygen delivery; **E_A_,** arterial elastance; **E_V_,** ventricular elastance; **FC**, fluid challenge; **HR**, heart rate; **LVEF**, lef ventricular ejection fraction; **MAP**, mean arterial pressure; **SAP**, systolic arterial pressure; **SV**, stroke volume; **TPRi**, total indexed peripheral resistance; ***VO_2_***, oxygen consumption.

| **1^st^ Component** | r | **2^nd^ Component** | **r** | **3^rd^ Component** | **r** |
| --- | --- | --- | --- | --- | --- |
| **E_A_** | 0.94 | LVEF | 0.72 | SvcO_2_ | 0.70 |
| SVRi | 0.78 | PAM | 0.60 | **E_A_/E_V_** | 0.54 |
| **E_V_** | 0.71 | **E_V_** | 0.58 | PP | 0.52 |
| HR | 0.52 | SW/PVA | 0.54 | PAM | 0.47 |
| MAP | 0.48 | PP | 0.44 | HR | 0.36 |
|  |  | SvcO_2_ | 0.26 |  | 0.28 |
| PP | -0.29 | GapCO_2_ | -0.41 | **E_V_** | -0.27 |
| SW/PVA | -0.63 | **E_A_/E_V_** | -0.61 | PVC | -0.40 |
| VES | -0.89 |  |  | LVEF | -0.44 |
|  |  |  |  | GapCO_2_ | -0.61 |

**Supplementary Figure 1**. eigen value of the fourteen principal component


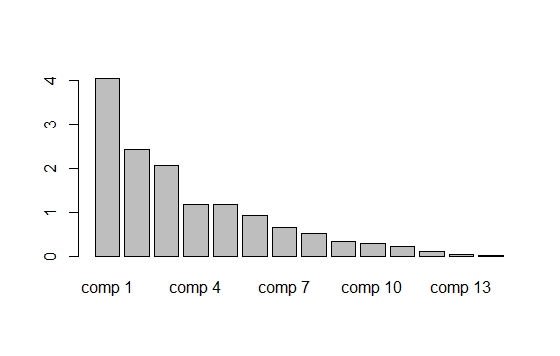


**Supplementary Figure 2**. Variable factors map for the first and third component. The 10 more contributory variable are represented.


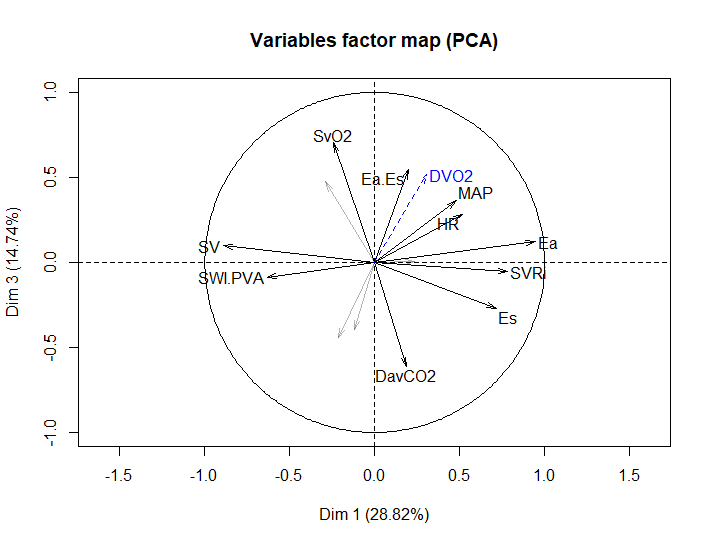

Supplement: Supplementary file 1 [file Data_Sheet_1.docx]
